# Transboundary spread of pig diseases: the role of international trade and travel

**DOI:** 10.1186/s12917-019-1800-5

**Published:** 2019-02-22

**Authors:** Daniel Beltran-Alcrudo, John R. Falco, Eran Raizman, Klaas Dietze

**Affiliations:** 1 Regional Office for Europe and Central Asia, Food and Agriculture Organization, Budapest, Hungary; 2 Animal Plant Health Inspection Service - International Service (USDA-APHIS-IS), United States Department of Agriculture, Riverdale, USA; 3 Animal Production and Health Division, Food and Agriculture Organization, Rome, Italy; 4 Institut für Epidemiologie, Friedrich-Loeffler-Institut, Greifswald - Insel Riems, Germany

**Keywords:** Transboundary animal disease (TAD), Swine diseases, Risk assessment, Informal trade

## Abstract

As globalization increases the interconnectedness between nations, economies, and industries, the introduction of diseases will continue to remain a prominent threat to the livestock sector and the trade of animals and animal products, as well as the livelihoods of farmers, food security and public health. The global pig sector, with its size and dichotomy between production type and biosecurity level, is particularly vulnerable to the transmission of transboundary animal diseases such as African and classical swine fever, foot and mouth disease, or porcine reproductive and respiratory syndrome. All of the above pose a constant threat to swine health, mainly as a result of both formal and informal international trade.

Inspired in the risk assessment methodology, this paper classifies and provides an overview of the different pig disease introduction and exposure pathways, illustrated with abundant examples. Introduction pathways are classified as formal international trade (by product), informal international trade (by product), and spread through fomites. Formal trade of pigs and pork products is regulated by legislation and measures protecting animal populations from exotic diseases. Much more difficult to control is the transboundary swine disease transmission originating through informal trade, which entails illegal smuggling, but also the informal cross-border transfer of animals and products for personal use or within informal market chains. Meat products are most commonly mentioned, although fomites have also played a role in some cases, with live pigs, being more difficult to smuggle playing a role less frequently. The main exposure pathways are also described with the oral route playing a prominent role.

Risk assessments can aid in the identification of pathways of pathogen introduction and exposure. However, quantitative information on informal disease introduction pathways remains very scarce and often incomplete, making it difficult to estimate the actual magnitudes of risks. Nevertheless, this knowledge is deemed essential to set up risk based awareness, prevention and surveillance programs that correspond to reality.

## Background

Livestock diseases adversely impact the productivity of livestock production systems by reducing the quantity and quality of livestock-derived products. Doing so, they do not only challenge the livelihoods of the producers, but can have major socio-economic consequences for the wider population. Additionally, the trade restrictions that often accompany the reporting of livestock diseases, may add a considerable economic burden, particularly to exporting countries. Control measures, such as massive depopulations, will also add to the cost, both from the economic and the social points of view. Moreover, zoonotic pig diseases must be judged by their impact on public health.

As examples of economic impact, the 1997 classical swine fever (CSF) epidemic in the Netherlands led to the destruction of 10 million pigs and an estimated cost of $2.3 billion US dollars [67]. The same year, foot and mouth disease (FMD) led to the destruction of Taiwan’s pig industry [86]. An even bigger economic impact was seen in the 2001 outbreak of foot and mouth disease virus (FMD) in the United Kingdom, which led to costs exceeding £3.1 billion ($4.4 billion) to the agriculture and food chain sectors alone [27, 113]. An example of the impact on livelihoods is exemplified by the 1978 African swine fever (ASF) epidemic in the Caribbean island of Hispaniola, controlled by the swine depopulation on the entire island, which had a particularly dramatic effect in the already precarious livelihood of the rural population of Haiti [70].

The larger the impact of animal diseases on agricultural production, the more evident the need to prevent the entry of pathogens into a country, and to ensure the preparedness of veterinary services and other stakeholders for timely detection and control. Transboundary animal diseases (TADs) are defined as those that are of significant economic, trade and/or food security importance for a considerable number of countries, which can easily spread to other countries and reach epidemic proportions, and where control/management, including exclusion, requires cooperation between several countries [38]. There is a long history of pig TADs entering new territories, in some cases through legal trade. One of the first documented examples dates back to 1887, when CSF was carried in a shipment of boars from England to Sweden [14].

With the ongoing process of globalization, the increasing movement, both formal and informal, of animals and their products poses a growing risk for animal populations to become exposed to TAD pathogens. A sound epidemiological understanding of how TADs may enter naive populations is essential to ensure preparedness of all stakeholders, to prevent the entry of pathogens through the different pathways, and to properly and promptly detect, investigate and control outbreaks. However, many countries, especially those in the developing world, lack adequate veterinary and diagnostic capacity to perform these actions. As a result, the source and pathways of primary or index outbreaks often remain unknown. This is perfectly exemplified by the latest ASF spread within the European Union and into China, where despite the high risk and alert levels, none of the last affected countries (Hungary, Romania, Bulgaria and Belgium) managed to identify or even narrow down the source of their index cases [128]. And yet, the source country or region can sometimes be narrowed down through phylogenetic analysis, which may point towards some more likely routes. For example, phylogenetic analysis of the UK isolate of the 2001 FMD epidemic, which started in a swill-feeding piggery, showed the greatest similarity to a South African virus [5, 94]. Strains of porcine epidemic diarrhea (PED) in the United States were almost identical with Chinese strains [56]. Similar examples for CSF have been compiled by Moennig et al. [68]. More recently, genetic analysis of the first ASF incursion in China proved its likely origin in Eastern Europe [128].

Within global livestock production, the pig sector plays a key role in the provision of animal protein. Pork represents the most consumed meat from terrestrial animals, accounting for over 36% of global meat intake, and its growth has been steady over the past decades [33, 40]. The pork producing sector is characterized by a marked dichotomy of production systems, demonstrated by traditional small-scale and subsistence-driven production on the one hand, and industrialized pig farming with increasing vertical integration of the value chain on the other [32]. Despite the progressive growth in importance of these larger scale operations to meet the growing global pork demand, a large proportion of farmers engaged in pig production (about 43% of all pigs worldwide), particularly in the developing world, operate in small-scale settings, and are often not linked to formal markets [99]. Pig rearing is a very common and traditional practice in rural areas, representing an important source of meat for the population in the countryside and often generating valuable cash income. Pigs are also valued for their quick turnover, prolificacy, and their ability to convert household waste into protein, their manure to fertilize fields and fishponds, and their usefulness as a financial safety net. However, small-holder pig rearing is usually a secondary income for households, which means that there is often little incentive or resources to improve housing facilities and biosecurity in general, or adopt husbandry-related technologies. It becomes evident that these very different stakeholder groups, i.e. commercial versus backyard, may not have the same priority in investing and adjusting production practices to disease prevention and control measures [45]. Indeed, the backyard sector, characterized by low biosecurity, outdated husbandry practices and technologies, and a poor awareness and compliance with animal health regulations (outbreak reporting, movement control, certifications, vaccination, etc.) plays a major role in the introduction, spread and maintenance of most pig diseases [24]. China, hosting around 49% of the world’s pig population, largely in low biosecurity systems, deserves particular attention [40, 118].

Despite the arguably important role played by backyard and free ranging pigs in the transmission of diseases, the sub-sector usually does not receive much institutional support to deal with animal diseases. Its development is often neglected by national authorities, who tend to concentrate their efforts on other livestock species or on the large-scale, commercial pig sub-sector. This means that the links between veterinary services and backyard smallholders are often missing or very weak. In some cases, there is a lack of trust or cultural barriers between farmers and the veterinary services. As a result, coupled with the frequent absence of a farm/animal identification and traceability system, and the limitations of veterinary services in terms of personnel, equipment and funding, the engagement of national authorities (veterinary services in particular) with smallholders is often insufficient, and ultimately challenges the implementation of disease prevention and control activities [11].

Transboundary swine diseases of major concern include ASF, CSF, porcine reproductive and respiratory syndrome (PRRS), FMD, and mosre recently, the upsurge of PED. They all have shown potential to spread across borders in the recent past, yet details regarding how exactly the spread between countries occurred remain missing [45, 93]. Like other livestock diseases, pig TADs can spread through the movement of live animals, but most importantly pig derived products, particularly pork, but also manure, semen, embryos, hides, etc. Furthermore, fomites such as contaminated trucks and animal feed can also play a role. In all cases, human behavior remains the underlying cause.

We must point out that, in an occurrence of multi-host diseases such as FMD, the pathway of spread may include the trade of animal species and products other than pigs and pork. This includes the trade (legal and illegal) of wildlife, their meat (e.g. bushmeat) and even their parasites [58]. The movements and migrations of wild suids (e.g. wild boar or warthogs) or other wild species across borders can also play a role, as exemplified by FMD in Bulgaria or ASF in Eastern Europe [4, 88]. However, this paper focuses on the spread via trade and will not consider these movements of wildlife, but just their role as potentially exposed hosts once the pathogen is introduced into a country via trade. Similarly, agro-terrorist events, i.e. the intentional introduction of pathogens, are not covered.

In order to properly prevent, prepare for, and address pig TAD incursions, risk assessment provides a structured and science-based process to assess the risk of such an event happening. Such risk assessments need to be timely and clearly shared with those involved in risk communication and management. Yet, the quality of the results from risk assessments heavily depends on the quality of the input data. Unfortunately, the spread of TADs occurs mainly though informal means, for which good quality data is scarce.

## Review

In this paper, we review the pathways for the transboundary spread of swine diseases via international trade and travel, inspired by the risk assessment process, looking into 1) introduction pathways, i.e. the pathways necessary for an importation activity to introduce pathogens into a particular country or region; and 2) exposure pathways, i.e. the pathways necessary for exposure of pigs in the importing country [85]. Table 1 compiles all pathways and references described in this paper.

**Table 1:**
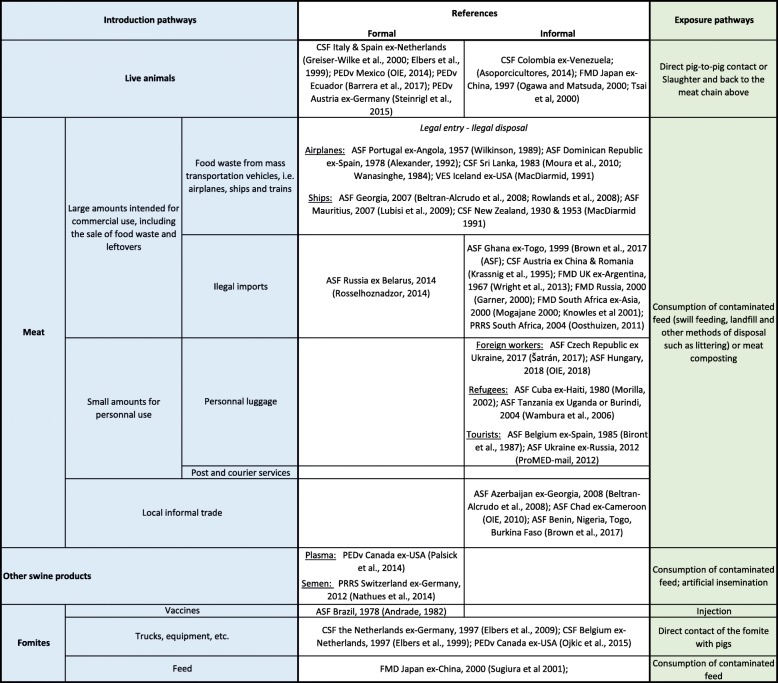
Main introduction and exposure pathways for transboundary swine diseases into free areas

## Introduction pathways

Within the introduction pathways, we have considered both formal and informal trade, each of which can be further subdivided according to the nature of the product, i.e. live animals or animal products.

### Formal international trade

Within the context of formal international trade, trading partners should follow the guidance and rules adopted by the authorities of the involved countries. If executed under the agreement of Sanitary and Phytosanitary Measures (SPS) of the World Trade Organization (WTO), animal health related trade requirements will be based on standards provided by the World Organization for Animal Health (OIE). These standards, set with the aim to protect animal and consumer health of the importing country, are a powerful tool to moderate market access and negotiating power. However, they may lead to disadvantages through high trade barriers for countries that are not capable to comply with these emerging requirements, or to voice their opinions and needs strong enough within the international community [44, 52, 74, 127]. The prominent role of animal health in international trade regulations leads to restrictive measures preventing the transboundary spread of pathogens. These regulations have great strength when looking at “known” threats and pathways of introduction, but are less effective at preventing informal movements or unknown pathogens. Nevertheless, history has shown that even within formal trade arrangements, the transboundary spread of swine diseases cannot be excluded with certainty [2]. Indeed, a number of qualitative risk assessment on the risk of CSF and ASF entry through legal imports of pork, live pigs and wild boar into different countries in Europe concluded that the risks, although low, cannot be ignored [17, 65, 74].

Within formal trade arrangements, the risk of disease spread is determined by the capability of applied measures to restrict the entry of potentially infected animals, products or fomites, and to timely identify known pathogens. Monitoring protocols which are insufficiently timed to detect a known threat represent an entry point for pathogens. Trade is particularly risky when dealing with free trade areas, such as the Economic Community of West African States (ECOWAS), the Southern Africa Development Community (SADC), the Common Market for Eastern and Southern Africa (COMESA), the East African Community (EAC), the Eurasian Economic Union (EEU) or the European Union (EU).

#### Live animals

The movement of animals in the incubation stage of disease or asymptomatic carriers represent major challenges, particularly when the infection has not yet been detected in the country of origin and the country still maintains a disease-free status, or before there has been time to effectively implement a transport ban. Although it did not result in an outbreak, a recent example showcasing this limitation within the control mechanisms of live animal trade was reported in Lithuania in 2014, when the export of 1704 ready-for-slaughter pigs to Poland took place only two days before an ASF outbreak was detected on the same farm [106]. Similarly, CSF spread from the Netherlands to Italy and Spain through the shipment of infected piglets in 1997 from an export-collection centre, before a ban on transportation was established [37, 48].

PED’s rapid global spread was mainly due to the fact that the movements took place at a time when the threat had not yet been identified, or testing protocols had not been validated and incorporated into trade certificate requirements. Mexico and Ecuador claimed the legal movement of animals as the source of introduction [9, 83]. Also the Dominican Republic and Colombia [8] did not exclude the possibility of legal movement of animals as a potential source of introduction [84]. The disease was also soon detected in several countries in Europe. The first case in Austria was traced to the movement of infected pigs from Germany [108].

#### Meat and other swine products

The main hazards associated with trade in pork and pork products are the viral agents of FMD, CSF, ASF and swine vesicular disease (SVD) [41]. Pathogens like ASFV are particularly problematic due to their prolonged infectiousness in meat products [1]. This is exemplified by the repeated detection of ASF viral DNA within pork products imported into the Russian Federation from Belarus. Each time, the meat has been effectively traced back to a number of meat processing plants in Belarus [100]. Although the presence of ASF in Belarus prevents the exporting of pigs or pork products to most countries, the fact that both countries belong to the Eurasian Economic Union (EEU), hinders the implementation of border controls to prevent the crossing of infected products.

Swine products other than pork can also be involved in the spread through legal movements, like the 2012 introduction of PRRS into Switzerland in 2012, traced back to boar semen imported from Germany despite routine testing for PRRSV [77]. The infection in the originating boar remained unnoticed until the semen was already exported and PRRS had spread to several swine herds. Another example, although with no transboundary spread, took place during the 1997–1998 CSF epidemic in the Netherlands, when two artificial insemination centres became infected and provided potentially contaminated semen to as many as 1680 farms all over the country, out of which 36 became infected [37].

The recent upsurge of PED in the United States and its further spread into other regions represents an example of how a high impact viral swine disease could follow a legal pathway to spread across international boundaries. Although not conclusively determined, it has been speculated that the causative agent, a coronavirus, entered the US in 2013 via contaminated feed [28, 53]. The PED virus spread into several countries over the next few years. Canada’s first outbreak in 2014 was attributed to spray-dried porcine plasma (SDPP) used as a feed supplement [91].

### Informal international trade

Although formal trading activities will always entail some risk for the spread of diseases, the informal or illegal movement of animals and animal products from infected countries involve a much higher risk [74]. Both formal and informal trade are closely correlated, since restrictions on the formal trade of animals and animal products actually promotes informal trade. Although this effect can only be estimated, such restrictions leading to reduced market access can act as an incentive to smuggling. In spite of the presence of pig TADs and the subsequent border checks, trade restrictions, fines and other regulatory barriers, the natural flow of animals and products from pig producing areas to those with a demand for pork and higher prices, will continue to occur through informal means.

#### Live animals

Although the illegal long-distance movement of live pigs is not likely to be significant due to their size and relatively low value, one cannot forget that the more valuable wildlife, such as wild boar, can also be involved in illegal movements to supplement recreational hunting stocks. There is prove of illegal national translocations in both Australia [105] and the United States [124]. In Italy, wild boar from Central Europe have been imported, sometimes illegally, since the 1950s [43]. Illegal wild boar translocations have also been reported Spain, originating from France [42]. Even the number of legally imported wild boar can be quite high in Europe, e.g. 13,000 only in 2013 in France, and is not exempt of health risks [50], as assessed by Martínez-López et al. [65]. In 2017, Bulgaria seized a shipment of live wild boar for hunting purposes from Poland, where multiple ASF cases in wild boar have been reported (Alexandrov, personal communication).

#### Meat and meat products

Illegal importation of meat (as opposed to live animals) seems to be the most common route of introduction of transboundary pig diseases. The main hazards associated with trade in pork and pork products are the viral agents of FMD, CSF, ASF and SVD [41]. Transmissible gastroenteritis of pigs (TGE) and PRRS are also perceived as potential hazards, but have not really lead to disease outbreaks due to the importation of pork products [41], except for the 2004 PRRS outbreak in South Africa [89].

There are two main categories of illegal movements according to the motivation of the persons involved in the trade/import: 1) the import of larger quantities with commercial intentions, e.g. containers; and 2) the informal import for personal consumption, e.g. in personal luggage [51]. Still, the division between the two is not always that clear. For example, passenger baggage may still occur in quantities which suggest the intention of commercial use [125]. Illegal movements can occur through a wide range of pathways. Regarding smuggled meat, Wooldridge et al. [125] described four main transport modes, i.e. sea freight, air freight, air passenger baggage, and post (plus couriers), to which road transportation should be added.

In this review, we have used a classification that aims to combine the two classifications above: 1) The smuggling of large quantities for commercial intentions, including the illegal sale and usage of catering leftovers from planes, ships and trains; and 2) The informal import for personal consumption (in personal luggage or by post/mail).

##### Commercial intentions (large amounts)

When considering large quantities with commercial intentions, import is achieved often due to the deliberate misdeclaration or mislabelling of cargo shipments [7]. Loopholes exist that can be used to circumvent trade regulations and allow the entrance of unregulated food products through ports. In the United States (US) for example, where trade regulations are substantial, such loopholes have been identified by shipping of goods to undermanned ports, or through a country that is eligible to export food to the US, and stating that the intermediate country is the country of origin [80]. One example was the introduction of ASF into Ghana in 1999, which was believed to be due to swill feeding with pork from Togo although purposely mislabelled as from France to by-pass trade bans [19]. The 1967–68 FMD epidemic in the United Kingdom was sourced to infected bone marrow in lamb products imported from Argentina [126].

Wildlife meat may also represent an important source of infection, particularly when there is no proper control of the carcasses of hunted animals. This was demonstrated in 1993, when Austria reported CSF-positive samples of wild boar meat imported illegally from China and Romania [61].

Catering and other food leftovers from airplanes and ships have often been implicated in outbreaks of exotic pathogens. While the entry itself through international carriers may in fact be legal, the infraction takes place when such food leftovers get sold for swill feeding or improperly disposed against the airport or port waste management regulations (i.e. legal entry – illegal disposal; Table 1). African swine fever spread has provided many of such examples. It is almost certain that the long-distance spread of ASF from Angola to Portugal in 1957 took place as a result of uncooked waste food being fed to pigs near Lisbon airport [122]. Subsequent outbreaks in Cuba, Malta, Italy, Brazil and the Dominican Republic in the 70s were also attributed to feeding pigs on swill from ports or airports [3, 122]. The 1978 ASF outbreak in Brazil was linked to Spain or Portugal through a police officer at the international airport in Rio de Janeiro, who apparently collected leftovers from meals served on international flights and fed his animals [63, 71]. The 1968 ASF outbreak in the Italian island of Sardinia was probably caused through the introduction of contaminated waste from the port of Cagliari or the military airport used to feed pigs [73]. A more recent example took place in Georgia in 2007, when ASF entered through the Port of Poti in contaminated catering waste from an international cargo ship originating in Southeast Africa [12, 101]. Following this introduction, ASF has spread since throughout Eastern and Central Europe. Also in 2007, ASF most probably entered Mauritius through the harbour in the form of infected meat that ended up as illegal swill fed to pigs in one or more parts of the island [62].

However, the involvement of ports and airports are not limited to ASF. The outbreak of CSF in Sri Lanka in 1983 was also linked back to airplane waste, with primary cases occurring close to the international airport in Colombo [71, 121]. Furthermore, on two occasions, in 1930 and 1953, CSF was introduced into New Zealand through the feeding of ship garbage to pigs [64], and the introduction of CSF to Haiti in 1996 in the suburbs of Port-au-Prince may have been introduced by plane from Cuba [35]. The 2004 PRRS outbreak in South Africa was suspected to originate from uncooked swill from the Cape Town Harbor or the Cape Town International Airport, which was then fed to pigs as uncooked swill [89]. Similarly, the 2000 FMD outbreaks in pigs in Siberia, in the Russian Federation, were allegedly the result from feeding of contaminated meat from the port of Vladivostock, although the crossing of contaminated vehicles through the border with China was also considered [47]. Also in 2000, the FMD outbreak in South Africa, the first in livestock since 1956, were suspected to originate from illegally feeding pigs with garbage from a ship from somewhere in Asia [60, 69]. Finally, although the likelihood of importing vesicular exanthema of swine (VES) in meat is extremely low, there was a single reported outbreak in Iceland attributed to the feeding of garbage from the US air force base at Keflavik [64].

Trains or road transport have not provided any recent example of transboundary spread of pig diseases. Still, the rapid CSF spread during the mid-nineteenth century in the US was partly attributed to the development of railways [14].

##### Personal use (small amounts)

Passenger luggage was found to be the biggest contributor to the risk of all studied disease hazards into the United Kingdom [125]. The risk of introducing exotic pathogens through personal consignments is a continuous threat, since considerable amounts of animal products from countries where TADs are endemic are continuously introduced illegally in such way, posing zoonotic and animal health risks. The individual travelers might carry pork products with them, which may come into contact with susceptible hosts through swill feeding or improper disposal. Very often, this happens without them being aware of the risk of introducing TADs and other agricultural plagues, bringing into focus the great importance of awareness raising in travelling populations. Without further elaborating on the sociological aspects of the movement of people, it has to be understood that they represent a rather heterogeneous group. Refugees or displaced people, diaspora communities, foreign workers, and tourists are of great importance, but all with a different context regarding their motives for bringing goods along.

The number of international migrants worldwide reached 258 million in 2017. Of those, the total number of refugees and asylum seekers in the world was estimated at 25.9 million in 2016 [115]. These millions of people tend to carry cheap and locally available food when they travel between countries or mail and receive parcels with food items. Tourism is similarly increasing and reached 1322 million arrivals in 2017 [116].

Foreign workers were implied in the first introduction of ASF in 2017 into the Czech Republic, which has been attributed to contaminated food (probably bacon) brought by Ukraine workers of a local hospital laundry, which was then accessed wild boar [102]. The same pathway was described in 2018 in Hungary’s first ASF wild boar case, believed to be due to food waste introduced by foreign citizens working in large numbers in industrial facilities around the introduction location [86].

Refugees and illegal immigrants can also play a role. Civil unrest leads to the displacement of people, who often attempt to take their belongings, including their livestock and food with them. Indeed, ASF may have entered Cuba in 1980 through food products brought by illegal Haitian immigrants [104]. Subsequently, the influx of refugees from both Cuba and Haiti were considered as a risk for ASF introduction into the US [66]. An ASF outbreak in Santa Catarina State, Brazil, was linked to war refugees from Angola who landed by boat bringing infected pigs [71]. Also in Tanzania, ASF outbreaks were believed to originate from pigs introduced into refugee camps from Uganda or Burundi [120].

As for the involvement of tourists, the 1985 ASF outbreak in Belgium was attributed to a neighbour of the index farm who brought home some pork from a tourist trip to Spain and fed the remnants to a local boar [16]. It was also speculated that the source of the first ASF outbreak in Ukraine could be due to tourists from the Russian Federation coming to Ukraine on holidays with meat products [96].

An extra sub-category would be the mailing through international postal services and courier companies such as FedEx and DHL, identified as one of the four main transport modes for smuggling meat by Wooldridge et al. [125]. Although we could not find concrete examples in the literature, the risk cannot be ignored, and indeed this pathway was considered as a major risk of ASF entry into the United States [20, 22].

Several studies have assessed the risks of disease introduction from illegally imported meats for a number of pig diseases, e.g. FMD, ASF, CSF, SVD and PRRS [18, 22, 23, 51, 55, 79, 125]. The characteristics and volumes of informal movements are, by definition, unknown. This forces epidemiologists and risk analysts to work with very limited quantitative data and to include informed guesses, estimates and proxies in their calculations leading to high levels of uncertainty. Several studies have estimated the amounts of illegally imported meat (including bushmeat) brought by individual travellers by extrapolating the frequency and quantities of seizures of illegal meat at the ports of entry, most often international airports [13, 21, 22, 26, 39, 57, 103, 111]. These studies helped increase the understanding of informal movement of animal products calculating theoretical amounts of 2800 tons (Frankfurt airport, Germany), 3287 tons (Charles de Gaulle airport, France), 11,875 tons (all United Kingdom) and 1013 t (all Switzerland) entering per year [13, 21, 39, 51]. The US Customs confiscated over 68,000 products and specimens derived from pigs between 2012 and 2016 [20]. The species of origin of the meat is not usually identified though. Illegally imported wildlife products must also be considered, particularly wild boar meat. According to the US Fish and Wildlife Service of all confiscated wild suid products during the 2006–2016 period, warthog products represented over 60% of confiscations followed by wild boar, bush pigs, unspecified swine products, and babirusa [20]. Wild boar meat has also been reported elsewhere [95]. However, the systematic check of luggage or parcels is not a practical, cost-effective, sustainable or socially acceptable approach, and authorities try to address the problem through demanding travelers’ signed declarations that they are not bringing food products, random (or preferably risk-based) luggage/parcels checks, dogs trained to sniff agricultural products, and deterring fines when such products are found. The most commonly used and cost-effective prevention measure is to use awareness campaigns directed to travelers on the risks involved by transporting meat and other agricultural products.

#### Local informal trade

Unlike the usually longer disease jumps described above, the movement of diseases through the less studied local traffic across international land borders requires to be treated separately due to some of its unique features. The local cross border traffic of people and goods follows different patterns than the traffic using mass transportation vehicles such as trains, ships, airplanes or long distance bus connections. The latter all have in common defined departure and arrival points, rather than following individual choices, which allows for the tracking of routes and transport volume. All studies known to the authors that give indications on volumes of informal meat importations, further discussed below, focused on personal luggage through mass transportation means.

It has to be understood that these land borders are often not more than an administrative demarcation line with little or no impact on local traffic for communities in border regions, including the movement of products and animals. However, border controls can create significant price differentials for agricultural products like meat, which will stimulate clandestine movements across the borders [90]. Local cross border traffic will mostly follows these price differences between countries, triggering active value chains. This can be further aggravated in the presence of diseases, which have a direct impact on prices. Additionally, cultural aspects such as nomadic and transhumant movements or pilgrimages, or the presence of the same ethnic communities at both sides of the border are also important factors.

A specific example of the spread of pig diseases through movements of communities of the same ethnicity was the sole ASF outbreak in Azerbaijan in 2008 in the village of Nic, about 180 km east of the Georgian border. The village has a majority of Christian inhabitants of Georgian origin who most likely visited relatives in neighboring Georgia bringing some pork products back [12].

The 1990s ASF epidemic in West Africa is the perfect example of how informal small trade between neighbouring countries can quickly spread a disease within a region [19]. The disease was introduced in the region in Côte d’Ivoire in 1996, affecting pigs fed on food waste from a skip in a suburb inhabited by many diplomats and other expatriates [36]. The disease then spread to Benin near a market where merchants of the entire sub-region gathered. Nigeria, Togo, Ghana and Burkina Faso followed in just a few years. In Togo, the source of infection was traced to pigs bought cheaply in Benin and disposed of in Togo [19].

Disease control measures themselves, as highlighted prior, can lead farmers to escape with their animals or to rapidly slaughter and sell the pork in their immediate neighbourhood and beyond. In 2010, the occurrence of ASF in northern Cameroon, outside the endemic area in the south, lead to the implementation of stamping out measures by the veterinary authorities. As a result, some farmers fled with their animals, crossing the Logone river to southwest Chad, spreading the disease with them [82].

The smuggling of pigs and pork products has been identified as the most likely introduction of CSF from Venezuela into Colombia in 2014, favoured by the existing price differential and exchange rates [97]. Likewise, the spread of the highly virulent variant of PRRS in south and Southeast Asia was likely driven by smuggling, as revealed from cross-border value chains studies [31]. Over the past few years, the ASF virus was detected in meat products confiscated at the border in Latvia (in six commercially packaged products from Belarus) and Hungary (in a pork meat sample originating from Ukraine) [34, 78]. These examples emphasize both the relevance of this transmission route and the difficulties it presents for prevention.

For island countries where land borders do not exist, informal small trade can still take place in a very similar manner through the use of boats. The 1997 FMD introduction into Taiwan was considered most likely to have resulted from smuggling pork or live animals from China by fishing boats [81, 114]. Fishermen had already been considered a high risk for ASF introduction into the US from Caribbean islands at the time when the disease was present in the region, since they were known to carry live pigs and pork products on board [66].

### Through fomites

Fomites are materials that can carry infection and can include vehicles, clothing, footwear, feed, or even vaccines. Insufficiently cleaned and disinfected empty livestock trucks that have been contaminated from transporting infected pigs have been recognized as an important route of disease transmission for pig diseases [30, 54]. Vehicles transporting pigs to farms, markets or slaughterhouses, delivering feed, or collecting carcasses represent a great risk for disease transmission [10]. Numerous studies have focused on the role of contaminated vehicles. The CSF introduction into the Netherlands in 1997 was traced to a transport truck that had been in contact with infected pigs or infectious material in Germany [37]. Afterwards within the same European epidemic, such trucks became the most important disease transmission route during the epidemic in the Netherlands [37, 107]. Also the 1997 primary CSF outbreak in Belgium was attributed to a transportation lorry from the Netherlands [37]. Canada suspected infected feeds and trucks coming from the US as the most likely way by which PED virus entered the country [87]. Contaminated vehicles from China have also been considered to be responsible for the PanAsia FMD strain outbreaks in pigs in Siberia in April 2000 [47]. This pathway has been further analyzed through risk assessments for CSF introduction into Denmark [17], as well as ASF [75].

Transmission by persons, i.e. in their shoes or clothes, is also a possible route of disease spreading. Although less likely due to the more limited amounts of contaminated materials that can be carried, the travelling time and distances usually involved, such pathway cannot be ruled out. Some profiles or professions pose higher risks, particularly those who own or visit pig premises, e.g. farmers, veterinarians, inseminators, pig dealers, etc. This was experimentally proven for CSF, when infections were recorded after persons visited susceptible pigs without changing clothing [98]. In the same way, one cannot forget the potential role played by wild boar hunters and others who get in close contact to wild boar, such as foresters, mushroom pickers, hikers, etc., who may carry the disease agent in their contaminated clothing and tools.

The spreading of CSF by contaminated instruments and drugs is also a possibility. Although the authors could not find any example of international spread, this route was demonstrated in Germany, during the 1971–1974 epidemic, when iatrogenic transmission was responsible for 38 outbreaks [119]. Even manufactured products like vaccines could potentially act as a vehicle in the spread of diseases. This was described in Brazil, when crystal violet vaccines against CSF, manufactured using samples from CSF-diseased animals, used ASF-infected animals instead, becoming implicated in the spread of the disease in Brazil [6].

Other contaminated fomites can include feed and other agricultural products. The entry of PED in feed in the United States and Canada is a good example [28, 91]. Indeed, a recent study determined that most pig pathogens can survive in animal feed ingredients or products often imported into the US, particularly conventional soybean meal, lysine hydrochloride, choline chloride, vitamin D and pork sausage casings [29]. During the 2000 FMD outbreak in cattle in Japan, the virus was likely introduced through imported Chinese wheat straw [110]. There were similar examples, although at national level, when epidemiological investigations in Lithuania and Latvia suggested that fresh grass and seeds contaminated by secretions from ASF-infected wild boar could be a possible source of infection for backyard pigs [49].

The accidental importation of biological vectors, like ticks of the *Ornithodoros* genus that can transmit ASF, is also a potential pathway, although very unlikely and never described.

### Exposure pathways

Whether it is through the formal or informal routes described, the introduction of pathogens into new territories does not in itself automatically lead to an outbreak. The necessary additional step of the pathogen coming into contact and successfully infecting a susceptible host must also occur. In this context, countries with large numbers of pig holdings with absent or minimal biosecurity (as seen in most traditional/backyard farming systems within the developing world) have a higher likelihood of foreign diseases entering the pig sector and becoming established [22, 51]. Studies looking at the risk of westward ASF spread into Eastern Europe placed the highest risk for introduction in those countries with large numbers of pigs in low-biosecurity premises [23, 59]. Risk increases with reductions in biosecurity, e.g. free-range or scavenging, poor cleaning and disinfection practises, swill feeding, absence of quarantines, etc., but also in countries with important wild boar populations.

The following main exposure pathways can be considered: 1) Direct pig-to-pig contact; 2) Feeding on contaminated meat; 3) Fomite-pig contact, e.g. vehicles. Bellini et al. [10] described the preventive measures available to reduce such exposures, specifically for ASF.

From the literature, it appears that the most outstanding biosecurity gap is the practice of feeding of susceptible hosts on infected products that have no undergone previous heat treatment. Such a pathway for the transmission of pig diseases, such as CSF or ASF, has long been acknowledged and experimentally proven [15]. It can occur through swill feeding, scavenging on open landfills and other methods of disposal such as littering [125].

The source of the infected meat often has its origin in another critical breach of biosecurity. As reviewed in the previous section, catering waste from ships, aircrafts, trains or buses, which should be destroyed or correctly disposed, are instead dumped and/or illegally sold and utilized as swill. There are numerous examples of such incursions into countries as detailed earlier.

Illegally imported meat products will be distributed in different ways depending on the intentions of the individual importing them. In some cases, the imported meat product may be intended as animal feed. However, most times, it is intended for human consumption, in which case the meat will be consumed directly at home, indirectly via retailers, or in catering establishments [51]. Surely, the human consumption of food contaminated with foreign pathogens happens on a daily basis in any given country without causing any outbreak. For example, Corso [22] assumed that only 15% of disease agents would survive handling in a household prior to being discarded in the USA. For an outbreak to happen, the untreated leftovers should reach susceptible species, either through swill feeding, improper disposal or via the contamination of fomites.

Swill feeding remains a recurrent reason for primary outbreaks in countries where it is allowed, despite legislation requiring swill to be cooked prior to feeding it to pigs [35]. Indeed, it has been highlighted as the most likely pathway to introduce exotic swine diseases into the USA and the Netherlands using simulation models [22, 55]. Numerous examples abound highlighting the hazards of feeding swill containing uncooked waste pork and pork products. Such lapses in biosecurity have been tied to the putative origin of the CSF epidemic in Europe from 1997 to 1998, which began in Germany in January 1997 [35]. Three primary CSF outbreaks in the 1986 epidemic in the UK were attributed to the feeding of unprocessed swill [123], and so were the CSF outbreaks in Switzerland in 1993, in Bulgaria, Germany and Poland in 1994 and in Austria in 1994 and 1995 [41]. The first ASF outbreak in the Netherlands was diagnosed on a farm near The Hague, illegally feeding swill from hospitals, hotels and restaurants [112].

The improper disposal of food waste is the other main pathway by which contaminated meat and other products may end up contact with pigs and wild boar. Un-regulated or un-fenced garbage disposal sites are open to invasion by wild boar or free-ranging pigs, who often use these sites to scavenge leftovers and food waste. Some of the first ASF outbreaks reported in Spain in 1960 were related to pigs scavenging in landfills (Sánchez-[117]). Picnic leftovers present a similar scenario. The ASF index cases in wild boar in the Czech Republic, Hungary and Belgium in 2017–18 likely followed one of such routes, although the precise pathway was never discerned. During the 1971–74 CSF epidemic in Germany, one infection in wild boar was traced to garbage from American barracks and slaughter offal used as bait during the hunting season [119]. Moreover, during the same epidemic, pigs were infected by feeding the offal of wild boar carcasses left by the hunters.

Contact may also arise through the use of meat compost on fields, which has also been shown as a possible route of transmission if the composting process does not adequately kill viral particles contained in the meat [46].

Live imported infected pigs may lead to outbreaks by infecting directly additional animals at their destination site (e.g. receiving farm, live animal market), or once slaughtered and processed when the resulting meat or slaughter leftovers are improperly disposed or swill fed. Home-slaughtering is a particularly risky practice, since blood and other leftovers will likely contaminate the farm, hence easily infecting other susceptible animals on the premises. In Africa, it has been reported that whenever there are ASF outbreaks, in the absence of a compensation policy, farmers hurriedly sell the dying and in contacts pigs and their meat to salvage some income, for example in Uganda [72, 76, 92]. The same practice is suspected in China following the 2018 introduction [128]. This enhances disease spread across large geographical areas within or even across countries. Alternatively, when pigs die their carcasses may be then improperly disposed by the owners, unwilling to report the disease, which is a practice described as early as the 1910s [14]. Such behaviour was in fact responsible for some CSF cases in wild boar due to farmers who buried or dumped dead piglets in a nearby wood in 1951 in Germany [25], which has also been reported as a common practice in Georgia and Uganda [11, 72].

## Conclusions

Determining the sources of particular outbreaks is never easy, and is rarely proved beyond doubt [35]. As a result, the real route of introduction into countries is often not elucidated and remains hypothetical [109]. This is clearly reflected by OIE public disease reports. Of all immediate notifications and follow-up reports for ASF, CSF, PED and PRRS in WAHIS (*n* = 7169), the “source of the outbreak(s) or origin of infection” is only specified in 4.6% of outbreaks, with the rest being reported as unknown (74.9%), left blank (17.6%), under investigation (1.8%), or as a combination of sources (1%).

Addressing the transboundary spread of swine diseases is becoming more difficult due to the continuous growth of pig numbers and pork production, often in low biosecurity and/or unregulated systems, combined with increased global interconnectivity of people and products, many times in an informal manner. However, the limited amount of data available on informal activities (e.g. animal movements or meat smuggling) and on precise outbreak sources, makes it very difficult to quantify the risks of the different introduction paths.

The introduction of new diseases is usually unexpected, and farmers, field veterinarians and diagnosticians are frequently not familiar with its presentation, which often translates into delays both in the field reporting and the diagnosis. By the time the outbreaks are finally detected, time has passed, the disease has spread making it increasingly difficult to identify the primary or index case and to recall details that could elucidate the potential sources and pathways of introduction. This is often coupled with the fact that key information is often hidden due to the involvement of illegal behaviours, and potential lack of trust between farmers and official veterinarians.

Veterinary services in many countries are under-funded, under-staffed, under-equipped and demotivated. Few veterinarians are trained on how to properly conduct outbreak investigations, which are not conducted systematically. This is often due to the failure of the veterinary services to understand the value of such information.

In some cases, there may be a purposeful refusal by the authorities to disclose information that may negatively affect the country’s economy (due to trade restrictions) or international image. Regardless of the case, it is essential to increase the availability and quality of epidemiological data to better understand TADs transboundary spread. Current risk assessments looking into TADs spread are leading the way, in some cases extrapolating from the very limited information available. Risk assessments should focus on informal trade, which represents the widest knowledge gap. Nevertheless, information on outbreak sources and pathways is required to develop prevention, awareness and surveillance programs that correspond to the reality. In the case of formal trade, risk assessment will allow to develop additional protocols and regulations to fill in the loopholes of the system.

Apart from the improvements needed in many countries and regions at the institutional level to allow a functional public veterinary system and customs (i.e. border control) to roll out proper prevention, surveillance and detection programs, the role played by farmers and the public at large will always remain the key to success. At farm level, acknowledging that the animal keeper represents the front-line of disease prevention and early detection, strong emphasis must be given to biosecurity measures (allowing the individual farm to protect itself to the best degree possible from disease introductions), and on the importance of reporting any outbreak suspicions to the authorities. This must not only be supported by adequate training opportunities for farmers and people offering farm-related services, but should ideally be reflected as core responsibility for these stakeholders within the livestock development policy framework. Since some diseases may be first manifested in wildlife, the same applies to hunters, forest rangers and others.

## Abbreviations

ASF: African swine fever
COMESA: Common Market for Eastern and Southern Africa
CSF: Classical swine fever
DNA: Deoxyribonucleic acid
EAC: East African Community
EC: European Commission
ECOWAS: Economic Community of West African States
EEU: Eurasian Economic Union
EU: European Union
FMD: foot and mouth disease
OIE: World Organisation for Animal Health
PED: Porcine epidemic diarrhea
PRRS: Porcine reproductive and respiratory syndrome
SADC: Southern Africa Development Community
SDPP: Spray-dried porcine plasma
SPS: Sanitary and Phytosanitary Measures
SVD: Swine vesicular disease
TADs: Transboundary animal diseases
TGP: Transmissible gastroenteritis of pigs
UNWTO: United Nations World Tourism Organization
USA: United States of America
VES: Vesicular exanthema of swine
WTO: World Trade Organization
